# A Starch Molecular Explanation for Effects of Ageing Temperature on Pasting Property, Digestibility, and Texture of Rice Grains

**DOI:** 10.3390/foods14152661

**Published:** 2025-07-29

**Authors:** Enpeng Li, Xue Xiao, Yifei Huang, Yi Ji, Changquan Zhang, Cheng Li

**Affiliations:** 1 Jiangsu Key Laboratory of Crop Genomics and Molecular Breeding/Key Laboratory of Plant Functional Genomics of the Ministry of Education/Jiangsu Key Laboratory of Crop Genetics and Physiology, Agricultural College of Yangzhou University, Yangzhou 225009, China; lep@yzu.edu.cn (E.L.); 19985702195@163.com (X.X.); 601202@163.com (Y.H.); lafeecao@163.com (Y.J.); cqzhang@yzu.edu.cn (C.Z.); 2 Jiangsu Co-Innovation Center for Modern Production Technology of Grain Crops, Yangzhou University, Yangzhou 225009, China; 3 Food & Nutritional Sciences Programme, School of Life Sciences, The Chinese University of Hong Kong, Hong Kong 999077, China

**Keywords:** rice ageing, starch molecular structure, pasting property, starch digestibility, texture

## Abstract

Alterations in rice qualities during ageing are related to changes in starch molecular structures. However, if and how storage temperature determines starch structure–function relations remain unknown. This study applied four storage temperatures to investigate the effects of ageing on starch structure–function relations. A small but significant variation was observed for starch chain lengths, and this variation depended on both rice varieties and storage temperatures. Rice grains aged at higher temperatures had much higher peak (~25% larger) and setback viscosities (~50% larger) compared to those stored at lower temperatures. The digestion rate constant was lowered (~10%) most significantly at 40 °C. However, the maximum starch digested percentage increased after ageing. All rice varieties showed the lowest hardness at 4 °C and the highest hardness at 40 °C (~20% larger) after ageing. The changes in starch molecular structures were consistent with altered rice properties according to the established structure–property correlations. These results could improve our understanding of the complex rice ageing process.

## 1. Introduction

The storage of freshly harvested rice grains under controlled conditions for a period of time is frequently performed as an insurance against poor yields and crop failure. A process called ageing occurs during the storage of rice grains, which can cause significant alterations in the chemical composition, pasting property, starch digestibility, and the texture of cooked rice [1,2,3,4]. For example, over the storage period, there may be a decrease in protein content [5], a degradation of the amylose short chains [6], a steady increase in the setback viscosity of rice flour [7], an obvious decrease in starch digestibility [2], and a hardening of the texture of cooked rice [8]. These structural, physicochemical, digestion, and textural alterations associated with rice ageing could be unfavorable to some consumers and favorable to others depending on individual preference and intended applications.

Numerous studies have been performed to understand the structural basis for the alterations in the starch physicochemical properties, the digestibility, and the texture of cooked rice after ageing [6,9]. For example, some studies have ascribed these changes to alterations in the rice chemical composition and to endogenous enzymatic reactions on rice starch, proteins, and lipids [10,11,12]. More recent studies have suggested that small but significant changes in starch molecular structures, such as chain-length distributions (CLDs), in a variety-dependent manner also contribute to the altered properties of rice grains during ageing [6,9]. For example, compared to fresh rice, aged rice has native and leached starch molecules with significantly smaller molecular sizes and average amylopectin chain lengths, which has been proposed to contribute to the lower pasting temperature and higher peak viscosity of aged rice flour, as well as to the higher hardness of aged rice grains [9].

Storage temperature is another critical factor in determining the effects of ageing on rice properties. For example, it has been shown that ageing did not change the starch digestibility of Jasmine brown rice after storage at an ambient temperature for 7 months [13]. However, another study showed that rice stored at 37 °C demonstrated slower starch digestibility than rice stored at 4 °C after storage for 12 months [3]. This suggests that ageing could significantly change the starch digestibility of cooked rice, but the effects may depend on the storage temperatures. However, the questions of if and how storage temperatures change starch molecular structures, as well as their relationship with rice pasting and textural properties (eating properties) and digestibility (health property), are currently less clear. A better understanding of such relations is necessary in order to determine the molecular mechanisms underlying the effects of ageing on rice properties. This could aid in the development of a scientifically sound means of rice storage, intended to maintain the eating and health properties of rice for as long as possible.

Therefore, this study aimed to investigate the effects of ageing temperature on the relations between the starch molecular structures, pasting property, digestibility, and texture of cooked rice. To this end, three common rice varieties from China were selected and stored under various temperatures (−20 °C, 4 °C, room temperature (RT), and 40 °C) for 30 days, 100 days, and 200 days. The change in starch molecular structures was characterized by size-exclusion chromatography and fluorophore-assisted capillary electrophoresis. The pasting property of rice flour was analyzed with a rapid viscosity analyzer. The texture of cooked rice was analyzed by a textural analyzer, and the starch digestibility of cooked rice was analyzed via an in vitro simulated human upper gastrointestinal digestion assay. The results from this study could help us to better understand the rice ageing process.

## 2. Materials and Methods

### 2.1. Materials

Three rice varieties were used in this study, including Nangeng (abbreviated as NG, japonica), Yangdao (abbreviated as YD, indica), and Yongyou (abbreviated as YY, hybrid of japonica and indica). All three rice varieties were grown and harvested together in Jiudian county, Yangzhou, Jiangsu, China, in 2021, and no biological replications were analyzed in this study. Rice grains were dried in an open space for 10 days and stored immediately for this study. Three rice grains were stored at four different storage temperatures: −20 °C, 4 °C, room temperature, and 40 °C. The storage periods were set at three time points in the experimental conditions: 30 days, 100 days, and 200 days. After storage, a thresher (TSL-150A, Dongguan, China) was used to husk the rice into brown rice grains; then, a miller (kett, yokyo, Japan) was used to turn the brown rice grains into polished rice grains. The rice flour was prepared by grinding the polished grains into powder using a cryo-grinder (MM400, Retsch, Haan, Germany), followed by sieving through a 100-mesh sieve. The moisture content of rice flour was measured by a moisture meter (HE53, Mettler Toledo, Zurich, Switzerland).

Porcine pancreatin and proteases from Streptomyces griseus (XIV type) were purchased from Sigma Aldrich Chemical Co. (St. Louis, MO, USA). D-glucose assay kit, isoamylase from Pseudomonas genus, and amyloglucosidase (200 units/mL) were obtained from Megazyme International, Ltd. (Bray, Co. Wicklow, Ireland). The total starch (AA/AMG) assay kit (Megazyme) was used for the analysis of total starch content. Dimethyl sulfoxide (DMSO, GR grade for analysis) and pullulan polysaccharide standards (peak molecular weight range from 342 to 2.35 × 106 Da) were obtained from Merck & Co., Inc. (Kenilworth, NJ, USA) and Polymer Standard Services (Mainz, Germany), respectively.

### 2.2. Total Starch and Protein Content

The total starch content of rice flour was measured according to the method described in the Total Starch (AA/AMG) Assay Kit (Megazyme). All the tests were carried out in triplicate. The total protein content of rice flour was analyzed using an element analyzer (Vario EL cube, Elemantar, Langenselbold, Germany), which could determine the nitrogen quantity in the samples. The conversion coefficient of nitrogen to protein was set at 5.95. The test was carried out in duplicate.

### 2.3. Starch Chain-Length Distributions

The starch chain-length distribution (CLD) was analyzed following a previous method with certain modifications [6]. Rice flour (~200 mg) was incubated overnight at 37 °C in tricine buffer containing protease (1.5 mL, 10 units/mL) to hydrolyze the protein. The mixture was then centrifuged at 4000× *g* for 10 min, and the supernatant and top light-yellow layer (hydrolyzed protein) were removed. The bottom white layer (starch) was rinsed three times with anhydrous ethanol and deionized water, respectively. The purified starch (10 mg) was dispersed in deionized water at 80 °C for 30 min. After being cooled down to 37 °C, rice starch was debranched by isoamylase in sodium acetate buffer for 3 h. The reaction was stopped by boiling in an 80 °C water bath for 60 min. The debranched starch was collected after freeze-drying overnight.

For whole-starch CLD analysis, debranched starch (8 mg) was dissolved in DMSO with LiBr (0.5% *w*/*w*) for 2 h. The samples were then analyzed by a LC-20AD SHIMADZU system coupled with three columns (precolumn, Gram 100, and Gram 1000, in sequence, PSS, Mainz Germany) and a RID-10A refractive index detector (SHIMADZU Corporation, Kyoto, Japan). A pullulan standard series (PSS), with peak molecular weight ranging from 342 to 2.35 × 106 Da, was used for calibration.

For amylopectin CLD analysis, debranched starch (0.5 mg) was first labeled with APTS (8-amino-1,3,6-pyrenetrisulfonic acid). The labeled samples were analyzed using an MDQ Plus FACE System (AB SCIEX, Framingham, MA, USA), coupled with a solid-state, laser-induced fluorescence detector and an argon-ion laser as the excitation source.

### 2.4. Pasting Propeties

Rice flour (2 g) was mixed with 20 mL distilled water; then, the suspension was analyzed by an RVA (rapid viscosity analyzer) (Newport Scientific, Warriewood, NSW, Australia), and the TCW (thermal cycle for Windows, V1) software was used to analyze the results [14,15]. The heating temperature rose from 50 °C to 95 °C at a rate of 5 °C/min; then, it was maintained at 95 °C for 7 min and decreased to 50 °C at a rate of 6 °C/min. The agitator rotated at a speed of 960 r/min for the first 10 s and then remained at 160 r/min. The viscosity value was expressed in cP (RVA viscosity unit).

### 2.5. Digestibility

An in vitro starch digestion assay was performed following a previous method with modifications [16]. Rice flour (100 mg) was mixed with distilled water (2 mL) and incubated in 37 °C water bath for 1 h. The in vitro digestion was started by adding 8 mL enzyme mixture solution (0.05 mg pancreatin and 2.5 mL amyloglucosidase in 0.2 M sodium acetate buffer (pH 6.0)). The digestion time points were set at 0 min, 5 min, 10 min, 15 min, 30 min, 45 min, 60 min, 90 min, 2 h, 3 h, 4 h, 5 h, and 6 h. At each time point, 0.1 mL of the aliquot solution was quickly transferred to 0.9 mL of anhydrous ethanol to stop the reactions. The digested glucose content was determined using the D-glucose assay kit . The digestibility data were fitted to the first-order kinetics via a nonlinear least squares (NLLS) refinement method according to a previous study [17], and two parameters were obtained: C_∞_ (the estimated percentage of starch digested after an infinite reaction time) and k (the coefficient of starch digestibility).

### 2.6. Textural Properties

Rice grains were cooked by a rice cooker with a rice-to-water ratio of 1:1.3, and the cooked rice was equilibrated at room temperature for 30 min. The textural properties of cooked rice were analyzed by a TPA (textural property analyzer) (TA.XT plus, Stable Micro Systems Ltd., Godalming,UK), following the method described in a previous study [18]. The hardness and stickiness values of each sample were analyzed 5 times.

### 2.7. Statistical Analysis

Mean values and standard deviations were calculated by SPSS (IBM, New York, NY, USA, V25). Analysis of variance (ANOVA) with Tukey’s pairwise comparisons at *p* < 0.05 were used to analyze the significance of the differences.

## 3. Results and Discussion

### 3.1. Starch and Protein Content

The starch and protein content of rice grains before ageing were about 88% and 7% (Table 1), respectively, which are consistent with the literature [19]. There was no clear alteration in the starch content over the ageing period, which is consistent with the findings of previous studies [4,10]. However, the protein content decreased, especially after 200 days’ ageing. This is consistent with a previous study, which showed that the amount of prolamins and glutelins decreased with the ageing of rice grains [5].

### 3.2. Starch CLDs

**Figure 1:**
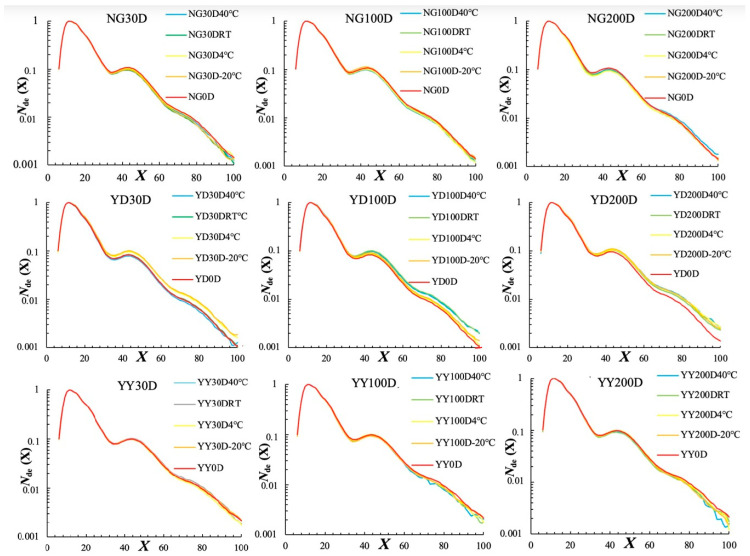
Amylopectin CLDs for different rice grains aged at different temperatures for different times. All CLDs were normalized to their peak maximum for an easy structural comparison. Note that 30D, 100D, and 200D refer to storage times of 30, 100, 200 days, respectively, and 40 °C, RT, 4 °C, and −20 °C refer to the storage temperatures.

Amylopectin CLDs, which were all normalized to the peak maximum for an easy structural comparison, were first characterized via fluorophore-assisted capillary electrophoresis (FACE) (Figure 1). According to the amylopectin cluster model, the amylopectin chains can be divided into different categories, including A (DP 6–12), B1 (DP 13–24), B2 (DP 25–36), and B3 chains (DP 37–100) [20]. The percentage of each chain length category was thus calculated and summarized in Table S1 in the supporting information in order to observe the effects of ageing on the amylopectin CLDs. A small but significant difference in amylopectin CLDs was observed after ageing, especially for the YD rice variety (Figure 1 and Table S1). This is consistent with a previous study [6], and was probably due to the degradation of amylopectin molecules by the endogenous α- or β-amylase during storage [21].

**Figure 2:**
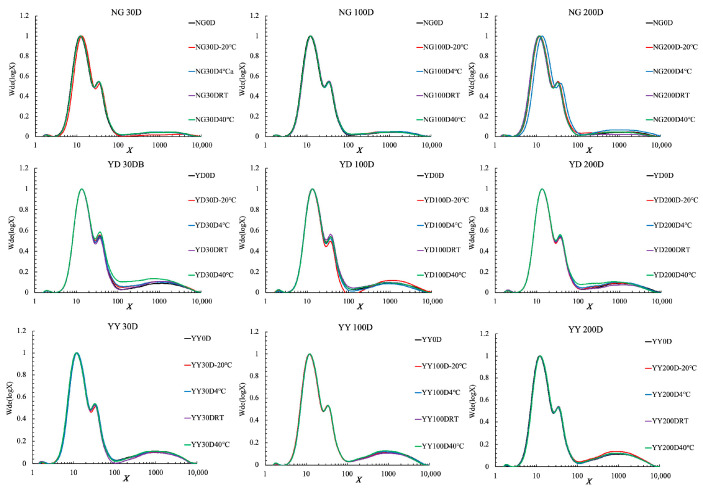
Whole starch CLDs for different rice grains aged at different temperatures and for different time periods. All CLDs were normalized to their peak maximum for an easy structural comparation. Note that 30D, 100D, and 200D refer to storage times of 30, 100, 200 days, respectively, and 40 °C, RT, 4 °C, and −20 °C refer to the storage temperature.

Similarly, a small but significant alteration occurred with the amylose CLDs (Figure 2). Amylose chains are frequently and arbitrarily defined as chains with DP above 100. The amylose contents for different rice grains can thus be calculated as the area under curve of DP > 100 divided by the whole CLD area (Vilaplana, Hasjim, and Gilbert, 2012), which is summarized in Table S2. In addition, the average chain length of the amylose chains was calculated and is shown in Table S2. The effects of ageing on the amylose CLDs depend on both the rice varieties and the storage temperatures. For example, a significant increase in amylose content was observed for NG rice grains after ageing for 200 days at 4 °C, which was, however, not the case for YD and YY rice grains. The average chain length of the amylose molecules was significantly decreased for NG rice grains after ageing for 200 days, while a decrease was not clearly observed for YD and YY rice grains. The significant change in the amylose chain length of NG rice grains is possibly due to its lower amylose content compared to the other two rice grain varieties, and the degradation of the amylose chains could thus have a profound effect on its chain lengths compared to the other two rice grains.

### 3.3. Pasting Property

The pasting parameters for differently aged rice flour varieties are summarized in Table 2, including the peak viscosity (PV), trough viscosity (TV), breakdown viscosity (BV), final viscosity (FV), and setback viscosity (SV). Interestingly, all the viscosity values increased after the ageing process, and the changes were found to be dependent on the storage temperatures. For example, rice grains aged at higher temperatures, such as 40 °C, had much higher PV and SV values compared to those stored at lower temperatures. This is consistent with the literature [4]. PV is generally determined by the water binding capacity and swelling powder of starch granules [22], and SV is determined by the retrogradation tendency of starch molecules during the short-term RVA cooling period [18]. This suggests that the ageing process has altered the water binding capacity and short-term retrogradation property of rice starch granules. The changes in the pasting property might be related to alterations in the starch molecular structures, as observed above, which will be explored in the following sections.

### 3.4. Starch Digestibility

The digestion parameters from the fitting of first-order kinetics are shown in Table 3, including the digestion rate constant and maximum starch digested percentage. The maximum starch digested percentage for the cooked fresh rice was about 82% to 85%, possibly due to the fully gelatinized starch granules [18]. Interestingly, the digestion rate constant for NG, YD, and YY was reduced by 45%, 18%, and 11%, respectively, after ageing for 200 days. Among the different storage temperatures, the digestion rate constant was lowered most significantly at 40 °C. However, the maximum starch digested percentage increased after ageing, suggesting that the digestion rate and extent are not necessarily positively correlated.

### 3.5. Texture

Textural attributes for different rice grains after ageing were measured by the texture analyzer and are summarized in Table 4. Hardness and stickiness are the most important textural attributes in determining the eating quality and consumers’ acceptance of cooked rice, although preferences for hardness or stickiness might depend on the region [23]. Among the three rice varieties, YY had the highest hardness value. This might be due to the higher amylose content of YY compared to NG and YD, as amylose molecules are prone to retrograde after cooking, contributing to the higher hardness value [19]. On the other hand, YD showed the lowest stickiness value compared to the NG and YY rice grains. The stickiness of cooked rice is generally determined by the amount and structure of starch and protein in the leachate during cooking [19]. This suggests that there was significant variation in the amount and structure of leached starch and protein in the different rice grains during cooking, which is consistent with a previous study [9].

A significant change in the textural attributes of the different rice grain varieties occurred after ageing, depending on the ageing temperatures and storage times. For example, all rice varieties showed the lowest hardness value at 4 °C, while they showed the highest value at 40 °C, which is consistent with the literature [4]. This is possibly due to the fact that higher ageing temperatures could decrease the leaching of the starch components, especially amylose [8]. Compared to hardness, the changes in stickiness were more complex and dependent on the rice varieties. For example, the stickiness of NG and YY decreased over the ageing period, especially at 40 °C, while the stickiness of cooked YD rice grains increased after 30 days’ ageing and then decreased after 100 days’ ageing.

### 3.6. A Starch Molecular Explanation for the Changes in Pasting Property, Digestibility and Texture During Ageing

**Figure 3:**
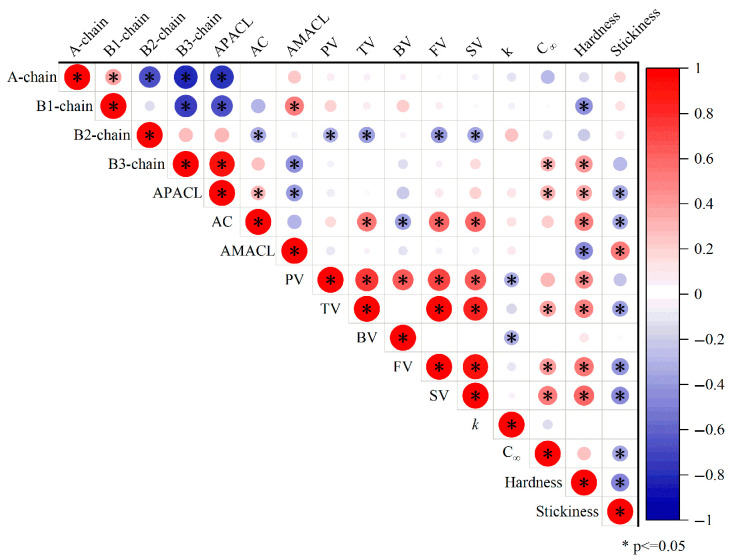
Pearson correlation coefficients among parameters of starch molecular structures, pasting property, digestibility, and texture of cooked rice.

The relations between the changes in the starch molecular structures, the pasting property, the digestibility, and the texture of cooked rice are extremely complex due to the inherently complicated process of rice ageing [4]. To further understand the mechanism that determines how ageing affects the starch digestibility of cooked rice, starch molecular structures were correlated with the pasting property, digestibility, and texture of cooked rice varieties (Figure 3).

Many correlations were found among the starch molecular structures, pasting property, digestibility, and texture of the cooked rice varieties. In terms of the pasting property, the amount of amylopectin B2-chains was significantly and negatively correlated with the peak viscosity, trough viscosity, final viscosity, and setback viscosity. This is different from a previous study, which showed that the amount of amylopectin intermediate chains (including B-2 chains) was positively correlated with the peak viscosity [17]. This difference could be due to the different samples used in different studies. In the study mentioned above, pure starches from different botanical sources were used. However, in the current study, rice flour was applied for the RVA analysis. This suggests that the relations between starch molecular structures and the pasting property could be greatly affected by the other ingredients in the samples. As commonly seen in the literature [16], the amylose content was positively correlated with the trough viscosity, final viscosity, and setback viscosity. This is because that amylose molecules are prone to retrograde due to their small molecular size and low branching degree, contributing to a high final and setback viscosity.

In terms of the starch digestibility, the digestion rate constant was negatively correlated with the peak viscosity and breakdown viscosity. This is expected, as a higher viscosity could potentially inhibit the access of starch molecules to the digestive enzymes [24]. On the other hand, the maximum starch digested percentage was positively correlated with the amount of amylopectin B3-chains, the average amylopectin chain length, the trough viscosity, the final viscosity, and the setback viscosity. The relations between the maximum starch digested percentage and the starch molecular structure/pasting property were different from those observed for the starch digestion rate. Again, this suggests that the digestion rate constant is not necessarily positively correlated with the maximum starch digested percentage. Higher amylopectin B3-chains and longer amylopectin chains are associated with a lower branching degree [25]. This could explain the positive correlation between the maximum starch digested percentage, the amount of amylopectin B3-chains, and the average amylopectin chain length; a higher branching degree could reduce the activity of starch-digestive enzymes due to steric hindrance [26]. The correlations between the maximum starch digested percentage and the viscosity parameters were not quite expected. Normally, the setback viscosity is negatively correlated with the starch digestibility, as the setback viscosity is contributed by the rapid retrogradation of amylose, which is a form of resistant starch [26]. Furthermore, the amylose content did not show any significant correlations with the digestion rate (*k*) or maximum digested percentage. Both of these results indicate that other structural factors (e.g., food matrix effect) could also affect the digestion of cooked rice, which could be examined in the future studies.

Texture might be another critical factor in determining the starch digestibility of cooked rice. However, the relations between the texture and digestion of cooked rice are complex; there are currently no universal conclusions. Hardness was negatively correlated with the amount of amylopectin B1-chains and the average amylose chain length (Figure 3), while it was positively correlated with the amount of amylopectin B3 chains, the average amylopectin chain length, the amylose content, the peak viscosity, the trough viscosity, the final viscosity, and the setback viscosity. It is understandable that the amount of amylopectin B1-chains was negatively correlated with hardness, while the amount of amylopectin B3-chains and average amylopectin chain length were positively correlated with hardness, as long amylopectin chains are possibly involved in short-term retrogradation [19]. Similarly, the negative correlation between hardness and average amylose chain length and the positive correlation between hardness and amylose content are probably due to the rapid retrogradation rate of amylose short chains [16]. The correlations between the hardness and the pasting parameters could also be explained by the contribution of retrogradation to the hardness. The relationships between stickiness, starch molecular structures, and pasting parameters were the opposite to those for hardness, which is reasonable as hardness is frequently negatively correlated with stickiness. Interestingly, for the first time, we showed that stickiness was negatively correlated with the maximum starch digested percentage, suggesting that a higher resistant starch content is related to sticker rice. Higher stickiness is frequently related to rice starch having a higher branching degree, which could supply more binding points to the compression probe of the texture analyzer [18]. This might explain the negative correlation between stickiness and the maximum starch digested percentage, as a higher degree of branching could inhibit enzymatic activity due to steric hindrance [27].

## 4. Conclusions

The current study investigated the effects of ageing temperature on the starch molecular structures, pasting property, digestibility, and texture of cooked rice. Minor but significant alterations were observed for the starch molecular structures, which were correlated with changes in the starch pasting property, digestibility, and texture of cooked rice. Typically, all viscosity values of rice flour increased after ageing, and a much higher peak and setback viscosity were observed when rice grains were stored at 40 °C. The digestion rate constant was significantly reduced for all the rice samples stored at 40 °C, while the maximum starch digested percentage increased after ageing. All rice varieties showed the lowest hardness value at 4 °C, while they showed the highest hardness value at 40 °C after ageing. These results indicate that 4 °C is the best storage temperature for maintaining the eating qualities of rice. Rice stored at 40 °C storage had the worst eating qualities; however, they had the slowest digestion rate.

## Figures and Tables

**Table 1:** Starch and protein content of rice grains with different storage times and temperatures.

	Ageing Time (d)	Ageing Temp (°C)	Starch (%)	Protein (%)
NG	0	/	87.10 ± 0.0 ^cd^	6.8 ± 0.0 ^ab^
	30	−20	86.68 ± 0.3 ^cd^	6.6 ± 0.0 ^bc^
		4	86.45 ± 0.2 ^cd^	6.7 ± 0.1 ^ab^
		RT	90.27 ± 0.5 ^ab^	6.9 ± 0.0 ^a^
		40	90.38 ± 0.1 ^ab^	6.6 ± 0.0 ^bc^
	100	−20	87.25 ± 0.0 ^c^	6.9 ± 0.1 ^a^
		4	85.88 ± 0.3 ^cd^	6.7 ± 0.0 ^bc^
		RT	89.99 ± 0.1 ^b^	6.6 ± 0.1 ^bc^
		40	90.32 ± 0.1 ^ab^	6.5 ± 0.1 ^c^
	200	−20	87.20 ± 0.6 ^c^	6.8 ± 0.0 ^ab^
		4	85.79 ± 0.3 ^d^	6.2 ± 0.1 ^d^
		RT	91.51 ± 0.4 ^a^	6.3 ± 0.1 ^d^
		40	90.86 ± 0.6 ^ab^	6.3 ± 0.0 ^d^
YD	0	/	88.3 ± 0.5 ^de^	7.2 ± 0.0 ^a–c^
	30	−20	90.1 ± 0.7 ^a–c^	7.2 ± 0.0 ^ab^
		4	88.7 ± 0.2 ^c–e^	7.2 ± 0.0 ^ab^
		RT	89.9 ± 0.3 ^a–c^	7.2 ± 0.0 ^b–d^
		40	89.7 ± 0.0 ^a–d^	7.4 ± 0.1 ^a^
	100	−20	90.1 ± 0.3 ^a–c^	7.3 ± 0.1 ^ab^
		4	87.9 ± 0.2 ^e^	7.2 ± 0.0 ^a–c^
		RT	89.1 ± 0.0 ^b–e^	7.0 ± 0.0 ^ed^
		40	90.2 ± 0.1 ^ab^	7.0 ± 0.0 ^ef^
	200	−20	88.7 ± 0.1 ^b–e^	6.9 ± 0.1 ^ef^
		4	87.8 ± 0.8 ^e^	7.1 ± 0.0 ^c–e^
		RT	89.6 ± 0.1 ^a–d^	6.9 ± 0.0 ^ef^
		40	90.7 ± 0.1 ^a^	6.8 ± 0.1 ^f^
YY	0	/	88.9 ± 0.3 ^a^	7.9 ± 0.0 ^a–c^
	30	−20	88.1 ± 0.1 ^ab^	8.0 ± 0.1 ^ab^
		4	86.5 ± 0.2 ^a–c^	8.0 ± 0.1 ^ab^
		RT	86.7 ± 0.0 ^bc^	8.1 ± 0.0 ^a^
		40	87.3 ± 0.3 ^ab^	8.0 ± 0.1 ^ab^
	100	−20	87.5 ± 0.6 ^ab^	7.6 ± 0.0 ^de^
		4	86.6 ± 0.6 ^bc^	7.8 ± 0.0 ^b–d^
		RT	86.6 ± 0.3 ^bc^	7.2 ± 0.0 ^fg^
		40	88.3 ± 0.0 ^ab^	7.7 ± 0.0 ^cd^
	200	−20	87.6 ± 0.7 ^ab^	7.5 ± 0.0 ^e^
		4	85.3 ± 0.4 ^c^	7.2 ± 0.1 ^g^
		RT	87.0 ± 0.6 ^a–c^	7.4 ± 0.1 ^ef^
		40	88.3 ± 0.5 ^ab^	7.5 ± 0.0 ^e^

Note: Means with different letters in the same column are significantly different at *p* < 0.05.

**Table 2:** Pasting properties of different rice grains with different storage times and temperatures.

	Ageing Time (d)	Ageing Temp (°C)	PV (cps)	TV (cps)	BV (cps)	FV (cps)	SV (cps)
NG	0	/	2957 ± 13 ^g^	1950 ± 124 ^f^	1007 ± 110 ^e^	2541 ± 136 ^f^	591 ± 115 ^d^
	30	−20	3694 ± 17 ^ef^	2127 ± 176 ^c–e^	1568 ± 159 ^cd^	2909 ± 183 ^c–e^	782 ± 112 ^bc^
		4	3359 ± 29 ^ef^	2011 ± 28 ^d–f^	1348 ± 1 ^d^	2798 ± 23 ^de^	787 ± 23 ^bc^
		RT	3810 ± 59 ^de^	2181 ± 78 ^c–e^	1629 ± 19 ^b–d^	2912 ± 78 ^c–e^	731 ± 21 ^c^
		40	4503 ± 114 ^c^	2428 ± 54 ^b–d^	2075 ± 60 ^ab^	3252 ± 50 ^cd^	824 ± 40 ^bc^
	100	−20	3739 ± 196 ^de^	2269 ± 77 ^c–e^	1470 ± 118 ^d^	3028 ± 107 ^cd^	759 ± 82 ^bc^
		4	3370 ± 141 ^ef^	1975 ± 125 ^d–f^	1396 ± 16 ^d^	2733 ± 128 ^e^	758 ± 111 ^bc^
		RT	4193 ± 90 ^cd^	2150 ± 11 ^c–e^	2044 ± 101 ^a–c^	2896 ± 26 ^de^	746 ± 8 ^bc^
		40	5217 ± 117 ^ab^	2939 ± 14 ^b^	2278 ± 131 ^a^	4001 ± 22 ^b^	1062 ± 11 ^a^
	200	−20	3369 ± 225 ^ef^	1990 ± 145 ^d–f^	1379 ± 110 ^d^	2730 ± 199 ^e^	740 ± 152 ^bc^
		4	3242 ± 24 ^f^	1861 ± 254 ^ef^	1380 ± 278 ^d^	2621 ± 212 ^e^	750 ± 220 ^a–c^
		RT	5086 ± 76 ^b^	2572 ± 4 ^bc^	2514 ± 71 ^a^	3414 ± 7 ^c^	842 ± 6 ^bc^
		40	5635 ± 32 ^a^	3573 ± 41 ^a^	2062 ± 72 ^a–c^	4708 ± 69 a	1135 ± 32 ^a^
YD	0	/	3918 ± 62 ^g^	2874 ± 39 ^f^	1144 ± 23 ^f^	4240 ± 39 ^g^	866 ± 22 ^f^
	30	−20	4657 ± 23 ^c^	3440 ± 71 ^ab^	1209 ± 94 ^c–e^	4708 ± 59 ^c^	1268 ± 53 ^d^
		4	4440 ± 19 ^c–e^	3043 ± 29 ^c–e^	1397 ± 10 ^b–d^	4392 ± 56 ^de^	1349 ± 42 ^d^
		RT	4580 ± 16 ^cd^	3330 ± 107 ^bc^	1250 ± 123 ^c–e^	4594 ± 60 ^cd^	1264 ± 90 ^d^
		40	5177 ± 32 ^a^	3448 ± 162 ^ab^	1729 ± 130 ^ab^	5078 ± 117 ^b^	1630 ± 113 ^c^
	100	−20	4377 ± 9 ^de^	3344 ± 1 ^bc^	1033 ± 8 ^de^	4670 ± 18 ^cd^	1326 ± 4 ^d^
		4	3926 ± 24 ^f^	2813 ± 124 ^e^	1113 ± 149 ^de^	4074 ± 134 ^f^	1261 ± 95 ^de^
		RT	4506 ± 94 ^cd^	2905 ± 15 ^de^	1601 ± 109 ^a–c^	4219 ± 97 ^ef^	1314 ± 46 ^d^
		40	4931 ± 83 ^b^	3343 ± 7 ^bc^	1587 ± 91 ^a–c^	5246 ± 31 ^b^	1903 ± 22 ^a^
	200	−20	4498 ± 31 ^cd^	2894 ± 41 ^de^	1604 ± 72 ^a–c^	4200 ± 69 ^ef^	1306 ± 59 ^d^
		4	4233 ± 53 ^e^	3313 ± 4 ^bc^	920 ± 48 ^ef^	4567 ± 2 ^cd^	1254 ± 2 ^e^
		RT	5046 ± 15 ^ab^	3133 ± 116 ^cd^	1912 ± 132 ^a^	4615 ± 104 ^cd^	1482 ± 89 ^cd^
		40	5131 ± 151 ^ab^	3717 ± 12 ^a^	1414 ± 164 ^b–d^	5549 ± 9 ^a^	1832 ± 10 ^b^
YY	0	/	2936 ± 6 ^g^	2497 ± 48 ^f^	539 ± 42 ^h^	3628 ± 14 ^f^	931 ± 17 ^e^
	30	−20	3682 ± 33 ^e^	2737 ± 182 ^c–e^	946 ± 12 ^fg^	4023 ± 168 ^cd^	1286 ± 141 ^bc^
		4	3299 ± 46 ^f^	2618 ± 66 ^de^	681 ± 44 ^gh^	3932 ± 91 ^c–e^	1080 ± 42 ^a^
		RT	3944 ± 75 ^d^	2852 ± 93 ^c–e^	1093 ± 18 ^d–f^	4029 ± 79 ^cd^	1177 ± 4 ^d^
		40	5099 ± 13 ^a^	3059 ± 30 ^bc^	2030 ± 43 ^a^	4557 ± 41 ^b^	1498 ± 50 ^b^
	100	−20	3691 ± 4 ^e^	2740 ± 19 ^c–e^	951 ± 16 ^fg^	3652 ± 0 ^e^	912 ± 4 ^e^
		4	3571 ± 44 ^e^	2554 ± 42 ^e^	1017 ± 86 ^ef^	3792 ± 51 ^de^	1238 ± 85 ^bc^
		RT	4276 ± 66 ^c^	2903 ± 10 ^cd^	1372 ± 55 ^cd^	4118 ± 64 ^c^	1215 ± 2 ^c^
		40	4775 ± 67 ^b^	3364 ± 161 ^b^	1410 ± 93 ^cd^	5123 ± 99 ^a^	1759 ± 32 ^a^
	200	−20	4042 ± 39 ^cd^	2515 ± 71 ^e^	1527 ± 110 ^bc^	3679 ± 69 ^e^	1164 ± 103 ^cd^
		4	4201 ± 15 ^c^	2802 ± 116 ^c–e^	1398 ± 101 ^cd^	3998 ± 113 ^cd^	1196 ± 93 ^cd^
		RT	4607 ± 68 ^b^	2781 ± 8 ^c–e^	1825 ± 60 ^ab^	4134 ± 16 ^c^	1353 ± 78 ^bc^
		40	5037 ± 142 ^a^	3733 ± 13 ^a^	1304 ± 129 ^c–e^	5327 ± 43 ^a^	1594 ± 93 ^b^

Note: Means with different letters in the same column are significantly different at *p* < 0.05. PV, peak viscosity; TV, trough viscosity; BV, breakdown viscosity; FV, final viscosity; SV, setback viscosity.

**Table 3:** Digestion parameters of different rice grains with different storage times and temperatures.

	Ageing Time (d)	Ageing Temp (°C)	*k* (min^−1^)/10^−3^	C_∞_ (%)
NG	0	/	17.41 ± 0.07 ^a–c^	84.70 ± 0.16 ^ef^
	30	−20	17.62 ± 0.14 ^ab^	89.40 ± 0.78 ^ab^
		4	17.92 ± 0.21 ^a^	90.07 ± 0.59 ^a^
		RT	17.49 ± 0.13 ^ab^	86.81 ± 0.08 ^c–e^
		40	17.41 ± 0.26 ^a–c^	87.34 ± 0.15 ^b–d^
	100	−20	15.63 ± 0.09 ^e^	87.15 ± 0.64 ^b–e^
		4	16.18 ± 0.12 ^de^	88.77 ± 0.15 ^a–c^
		RT	16.38 ± 0.27 ^c–e^	83.67 ± 0.11 ^f^
		40	16.73 ± 0.19 ^b–d^	83.39 ± 0.52 ^f^
	200	−20	10.70 ± 0.30 ^fg^	86.25 ± 1.12 ^de^
		4	11.11 ± 0.06 ^f^	86.73 ± 1.02 ^c–e^
		RT	9.74 ± 0.11 ^gh^	87.55 ± 1.01 ^b–d^
		40	9.49 ± 0.23 ^h^	89.60 ± 0.08 ^ab^
YD	0	/	16.88 ± 0.05 ^a^	83.18 ± 0.66 ^e^
	30	−20	15.61 ± 0.18 ^bc^	86.3 ± 0.17 ^d^
		4	14.90 ± 0.21 ^c–f^	88.94 ± 0.37 ^c^
		RT	15.42 ± 0.12 ^b–d^	87.21 ± 0.25 ^d^
		40	15.41 ± 0.09 ^b–d^	86.62 ± 0.61 ^d^
	100	−20	14.63 ± 0.21 ^f^	90.61 ± 0.03 ^b^
		4	16.07 ± 0.14 ^b^	91.04 ± 0.71 ^b^
		RT	15.42 ± 0.17 ^b–e^	90.76 ± 0.36 ^b^
		40	14.59 ± 0.08 ^f^	89.98 ± 0.20 ^bc^
	200	−20	14.90 ± 0.09 ^d–f^	90.88 ± 0.11 ^b^
		4	15.11 ± 0.11 ^c–f^	90.14 ± 0.06 ^bc^
		RT	14.74 ± 0.20 ^ef^	90.66 ± 0.18 ^b^
		40	13.81 ± 0.16 ^g^	92.62 ± 0.33 ^a^
YY	0	/	17.20 ± 0.01 ^c–e^	82.04 ± 0.06 ^e^
	30	−20	15.41 ± 0.09 ^i^	87.72 ± 0.54 ^cd^
		4	16.23 ± 0.21 ^gh^	88.03 ± 0.23 ^cd^
		RT	16.33 ± 0.14 ^fg^	87.66 ± 0.23 ^cd^
		40	15.42 ± 0.06 ^hi^	88.15 ± 0.24 ^c^
	100	−20	18.01 ± 0.12 ^ab^	87.96 ± 0.16 ^cd^
		4	17.87 ± 0.10 ^a–c^	90.01 ± 0.20 ^b^
		RT	16.94 ± 0.22 ^d–g^	92.08 ± 0.09 ^a^
		40	17.03 ± 0.09 ^d–f^	90.02 ± 0.23 ^b^
	200	−20	16.65 ± 0.11 ^e–g^	87.66 ± 0.57 ^cd^
		4	18.64 ± 0.14 ^a^	86.68 ± 0.44 ^d^
		RT	17.64 ± 0.21 ^b–d^	87.63 ± 0.06 ^cd^
		40	15.31 ± 0.08 ^i^	90.51 ± 0.63 ^b^

Note: Means with different letters in the same column are significantly different at *p* < 0.05.

**Table 4:** Textural parameters of different rice grains with different storage times and temperatures.

	Ageing Time (d)	Ageing Temp (°C)	Hardness (N)	Stickiness (N)
NG	0	/	9152 ± 81 ^cd^	1329 ± 5 ^ab^
	30	−20	8315 ± 113 ^e^	1346 ± 101 ^ab^
		4	8025 ± 133 ^e^	1108 ± 22 ^bc^
		RT	8410 ± 50 ^e^	1277 ± 23 ^a–c^
		40	9666 ± 71 ^bc^	1470 ± 181 ^a^
	100	−20	8634 ± 28 ^de^	1063 ± 51 ^b–d^
		4	8140 ± 78 ^e^	1001 ± 21 ^b–d^
		RT	9310 ± 3 ^b–d^	953 ± 71 ^cd^
		40	10,960 ± 215 ^a^	727 ± 8 ^d^
	200	−20	9711 ± 34 ^bc^	1111 ± 91 ^bc^
		4	9343 ± 65 ^bc^	1059 ± 79 ^b–d^
		RT	9959 ± 316 ^b^	1071 ± 2 ^b–d^
		40	11,297 ± 413 ^a^	748 ± 179 ^d^
YD	0	/	8985 ± 368 ^cd^	865 ± 90 ^cd^
	30	−20	9148 ± 12 ^cd^	1346 ± 101 ^a^
		4	8124 ± 19 ^d^	1108 ± 22 ^a–c^
		RT	9062 ± 157 ^cd^	1277 ± 23 ^ab^
		40	9355 ± 266 ^c^	1470 ± 181 ^a^
	100	−20	9450 ± 461 ^c^	719 ± 128 ^de^
		4	8750 ± 186 ^cd^	547 ± 39 ^de^
		RT	9664 ± 28 ^c^	843 ± 103 ^cd^
		40	11,646 ± 178 ^ab^	383 ± 80 ^e^
	200	−20	10,741 ± 4 ^b^	895 ± 47 ^b–d^
		4	9224 ± 40 ^c^	895 ± 171 ^b–d^
		RT	11,716 ± 6 ^ab^	826 ± 34 ^cd^
		40	12,666 ± 640 ^a^	872 ± 37 ^cd^
YY	0	/	10,656 ± 145 ^fg^	1057 ± 29 ^ab^
	30	−20	9894 ± 20 ^g^	1177 ± 191 ^ab^
		4	10,141 ± 10 ^g^	1021 ± 128 ^a–c^
		RT	12,361 ± 254 ^de^	1201 ± 40 ^ab^
		40	13,650 ± 65 ^bc^	1364 ± 25 ^a^
	100	−20	11,455 ± 250 ^ef^	980 ± 27 ^a–c^
		4	10,220 ± 314 ^g^	654 ± 68 ^cd^
		RT	12,913 ± 668 ^cd^	820 ± 135 ^bc^
		40	14,584 ± 417 ^ab^	289 ± 71 ^d^
	200	−20	11,582 ± 268 ^ef^	889 ± 134 ^bc^
		4	10,519 ± 10 ^fg^	650 ± 59 ^cd^
		RT	13,430 ± 89 ^cd^	837 ± 137 ^bc^
		40	15,125 ± 305 ^a^	369 ± 55 ^d^

Note: Means with different letters in the same column are significantly different at *p* < 0.05.

## Data Availability

The original contributions presented in the study are included in the article/Supplementary Materials. Further inquiries can be directed to the corresponding author.

## Supplementary Materials

The following supporting information can be downloaded at https://www.mdpi.com/article/10.3390/foods14152661/s1: Table S1: Amylopectin CLD parameters for different rice varieties after ageing.Table S2: Amylose CLD parameters for different rice varieties after ageing.
