# Supplementary material for: A Starch Molecular Explanation for Effects of Ageing Temperature on Pasting Property, Digestibility, and Texture of Rice Grains
Source: Foods. 2025 Jul 29;14(15):2661. doi: 10.3390/foods14152661 (PMC12346650; doi:10.3390/foods14152661)
Supplement: Supplementary file 1 [file foods-14-02661-s001.zip › foods-3749684-supplementary.pdf]

A starch molecular explanation for effects of ageing temperature on pasting property, digestibility and texture of rice grains

Table S1. Amylopectin CLD parameters for different rice varieties after ageing.

	Ageing time (d)	Ageing temp (℃)	A-chain (6 < DP ≤12)	B1-chain (12 < DP ≤24)	B2-chain (25 < DP ≤36)	B3-chain (36 < DP ≤100)	ACL
NG	0	/	28.23±0.89a	47.64±0.88a	10.72±0.83a	13.41±0.94a	20.94±0.53a
	30	-20	28.76±0.00a	48.31±0.19a	10.03±0.03ab	12.91±0.21a	20.66±0.09a
		4	28.75±0.16a	47.87±0.14a	10.09±0.03ab	13.29±0.29a	20.82±0.13a
		RT	28.90±0.40a	48.05±0.25a	10.14±0.08ab	12.91±0.57a	20.67±0.26a
		40	28.74±0.23a	47.95±0.01a	10.10±0.07ab	13.21±0.17a	20.79±0.09a
	100	-20	28.26±1.00a	47.92±0.34a	10.40±0.32a	13.43±1.01a	20.88±0.54a
		4	28.87±0.44a	48.29±0.44a	10.27±0.12ab	12.56±0.77a	20.53±0.36a
		RT	28.63±0.13a	47.91±0.08a	10.20±0.11ab	13.25±1.00a	20.81±0.02a
		40	28.91±0.51a	47.98±0.13a	10.10±0.08ab	13.01±0.27a	20.70±0.10a
	200	-20	28.55±0.48a	47.95±0.55a	10.27±0.18ab	13.23±0.84a	20.83±0.37a
		4	29.9±0.81a	47.90±0.05a	9.01±0.52b	13.19±0.51a	20.58±0.13a
		RT	29.12±0.32a	47.80±0.26a	9.85±0.10ab	13.24±0.48a	20.69±0.11a
		40	29.51±0.24a	47.69±0.31a	9.60±0.37ab	13.20±0.75a	20.85±0.20a
YD	0	/	29.54±0.94ab	48.42±0.41abcd	9.82±0.41ab	12.22±0.93bc	20.36±0.47abc
	30	-20	29.32±1.38ab	48.18±0.33abcd	9.85±0.42ab	12.65±0.29abc	20.66±0.66abc

		4	29.15±0.71ab	48.00±0.21abcd	9.86±0.15ab	12.97±0.34abc	20.69±0.23abc
		RT	29.94±0.21ab	48.067±0.05ab	9.52±0.08ab	11.85±0.24bc	20.17±0.10bc
		40	30.65±0.33a	48.79±0.16a	9.28±0.14b	11.26±0.16c	19.87±0.11c
	100	-20	29.45±0.60ab	48.55±0.04abc	9.88±0.14ab	12.11±0.42bc	20.35±0.22abc
		4	29.43±0.40ab	48.14±0.18abcd	9.77±0.00ab	12.64±0.58abc	20.64±0.28abc
		RT	28.04±0.54b	48.01±0.13abcd	9.90±0.11ab	14.04±0.30ab	21.23±0.20ab
		40	28.26±0.65ab	48.13±0.38abcd	9.85±0.31ab	13.75±0.72ab	21.09±0.37ab
	200	-20	27.48±0.64b	47.70±0.06d	10.29±0.21a	14.52±0.37a	21.52±0.22a
		4	27.71±0.08b	47.96±0.16cd	9.99±0.04ab	14.52±0.04a	21.51±0.00a
		RT	27.90±0.15b	47.91±0.07bcd	10.07±0.07ab	14.08±0.01ab	21.32±0.01ab
		40	27.53±0.25b	47.90±0.03bcd	10.00±0.16ab	14.55±0.38a	21.52±0.17a
YY	0	/	28.71±0.09ab	47.54±0.05a	10.02±0.05a	13.73±0.20ab	21.12±0.05ab
	30	-20	28.72±0.03ab	47.41±0.07a	9.91±0.11a	13.94±0.07ab	21.18±0.01ab
		4	28.87±0.06ab	47.37±0.11a	10.01±0.08a	13.74±0.26ab	21.10±0.05ab
		RT	28.18±0.01b	47.03±0.01a	10.06±0.00a	14.71±1.56a	21.61±0.06a
		40	28.99±0.33ab	47.90±0.80a	9.75±0.21a	13.35±0.91ab	20.75±0.20b
	100	-20	29.07±0.31a	47.54±0.47a	9.70±0.22a	13.67±0.44ab	20.98±0.22b
		4	28.43±0.20ab	47.57±0.04a	9.92±0.13a	14.09±0.29ab	22.15±0.08ab
		RT	28.91±0.40ab	47.58±0.20a	9.71±0.27a	13.79±0.33ab	20.96±0.10b
		40	28.85±0.34ab	47.87±0.07a	9.81±0.14a	13.46±0.27ab	21.04±0.35b
	200	-20	28.84±0.08ab	47.35±0.01a	9.77±0.14a	14.04±0.23ab	21.21±0.13ab

	4	28.50±0.05ab	47.67±0.40a	9.86±0.12a	13.97±0.23ab	21.25±0.02ab
	RT	29.18±0.13a	47.94±0.31a	9.76±0.00a	13.11±0.18b	20.78±0.06b
	40	29.09±0.30a	47.34±0.30a	9.71±0.14a	13.86±0.13ab	21.13±0.10ab

Note: Means with different letters in the same column are significantly different at $p < 0.05$.

Table S2. Amylose CLD parameters for different rice varieties after ageing.

	Ageing time (d)	Ageing temp (℃)	AC (%)	ACL
NG	0	/	7.86±0.14cd	1710±86b
	30	-20	4.19±0.06e	2339±21a
		4	8.37±0.62c	1533±2c
		RT	7.67±0.32d	1542±98c
		40	8.38±0.09c	1529±3c
	100	-20	8.18±0.4cd	1407±62d
		4	8.45±0.88c	1421±167cd
		RT	7.66±0.1d	1393±20d
		40	7.98±0.2cd	1325±37e
	200	-20	8.87±0.25c	1466±224cd
		4	10.53±2.14b	1514±3c
		RT	13.21±12.17a	1305±46e
		40	7.98±0.18cd	1406±113d
YD	0	/	15.59±1.44bc	1613±191bc
	30	-20	16.2±1.42b	1620±103bc
		4	19.04±3.42a	1515±53c
		RT	17.7±0.92ab	1712±216b
		40	12.84±11.19de	2064±1081a
	100	-20	18.59±2.05a	1735±543b
		4	10.73±3.12f	1549±215c
		RT	13.62±2.27de	1639±355bc
		40	15.96±0.44bc	1417±7d
	200	-20	16.65±1.84b	1491±116cd
		4	16.45±0.61b	1520±187c
		RT	14.36±2.89d	1500±138cd
		40	14.74±3.4d	1499±568cd
YY	0	/	18.25±0.9ab	1439±12b
	30	-20	16.16±0.7bc	1584±106a
		4	16.96±0.22bc	1500±55ab
		RT	15.8±1.61c	1509±64ab

	40	17.54±0.39b	1450±12b
100	-20	17.75±0.72b	1367±6c
	4	18.91±0.76ab	1378±8c
	RT	18.02±2.42b	1376±11c
	40	18.88±0.32ab	1287±53cd
200	-20	19.61±0.87a	1351±39c
	4	18.07±0.19b	1393±9bc
	RT	17.48±0.65b	1408±3bc
	40	18.59±0.45ab	1313±72c

Note: Means with different letters in the same column are significantly different at $p < 0.05$.
